# Supplementary material for: CipA mediates complement resistance of Acinetobacter baumannii by formation of a factor I-dependent quadripartite assemblage
Source: Front Immunol. 2022 Jul 26;13:942482. doi: 10.3389/fimmu.2022.942482 (PMC9361855; doi:10.3389/fimmu.2022.942482)
Supplement: Supplementary file 2 [file DataSheet_2.pdf]

Supplementary figure 2

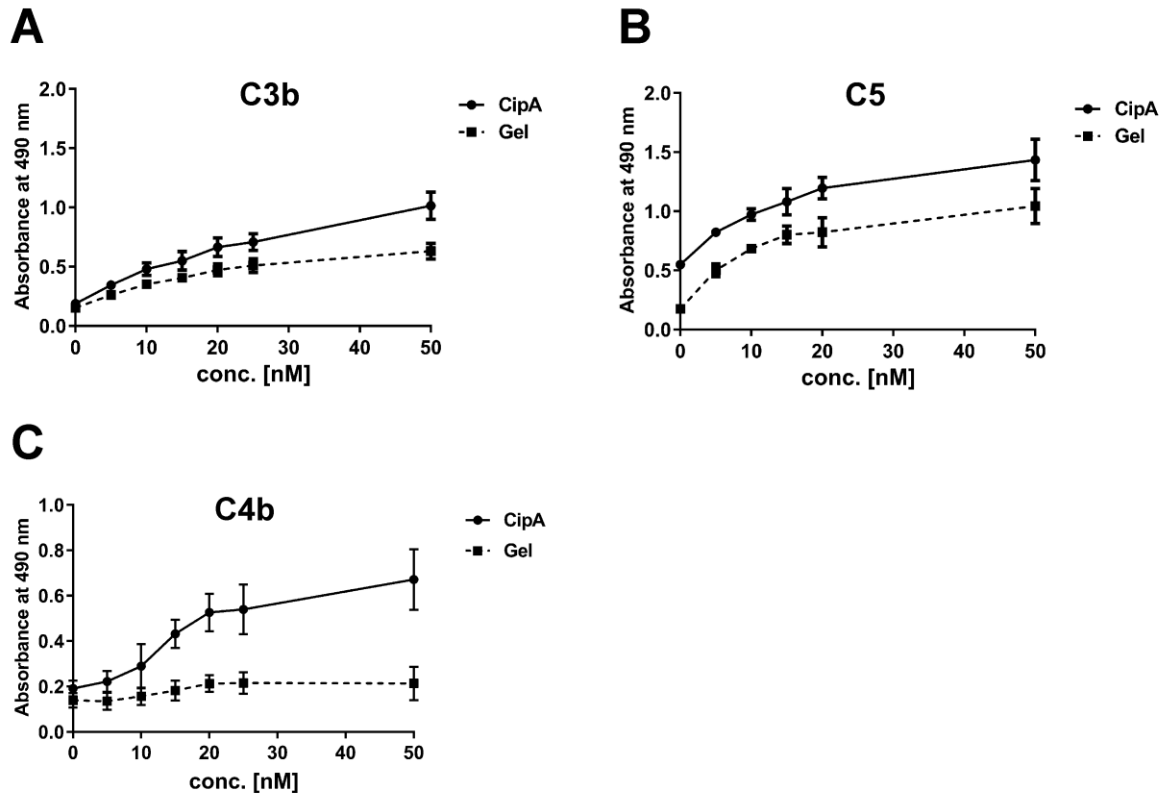

Dose-dependent binding of complement C3b, C5 and C4b to CipA. Binding of C3b **(A)**, C5 **(B)**, and C4b **(C)** to CipA was assessed by ELISA. CipA (5 ng/µl) was immobilized and incubated with increasing concentrations (0 to 50 nM) of purified complement components. Data represent means and standard deviation of at least three different experiments, each conducted in triplicate. Gel, gelatin.
