# Supplementary material for: CipA mediates complement resistance of Acinetobacter baumannii by formation of a factor I-dependent quadripartite assemblage
Source: Front Immunol. 2022 Jul 26;13:942482. doi: 10.3389/fimmu.2022.942482 (PMC9361855; doi:10.3389/fimmu.2022.942482)
Supplement: Supplementary file 3 [file DataSheet_3.pdf]

Supplementary figure 3

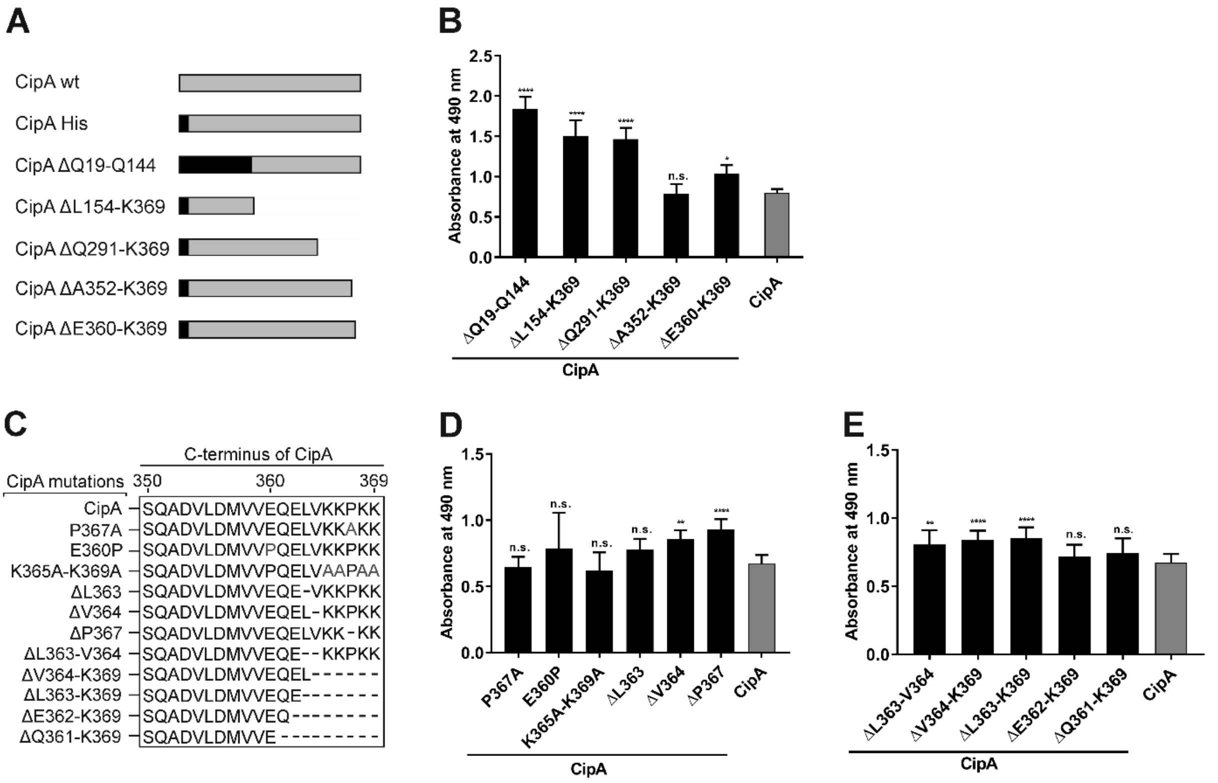

Binding of C3b to CipA variants. Schematic representation of N- and C-terminal deletion CipA variants **(A)** the introduced deletions and substitutions within the C-terminus of CipA **(C)**. Protein binding of C3b to CipA variants was measured by ELISA **(B, D,** and **E)**. CipA (5 ng/µl) coated wells were incubated with purified C3b (5 ng/µl) and antigen-antibody complexes were detected using an anti-C3 antibody (1:1,000). Data represent means and standard deviation of at least three different experiments, each conducted in triplicate. *, p ≤ 0.05; **) p ≤ 0.005 ****, p ≤ 0.0001, n.s., no statistical significance, one-way ANOVA with post-hoc Bonferroni multiple comparison test (confidence interval = 95%).
