# Supplementary material for: CipA mediates complement resistance of Acinetobacter baumannii by formation of a factor I-dependent quadripartite assemblage
Source: Front Immunol. 2022 Jul 26;13:942482. doi: 10.3389/fimmu.2022.942482 (PMC9361855; doi:10.3389/fimmu.2022.942482)
Supplement: Supplementary file 7 [file DataSheet_7.pdf]

Supplementary figure 7

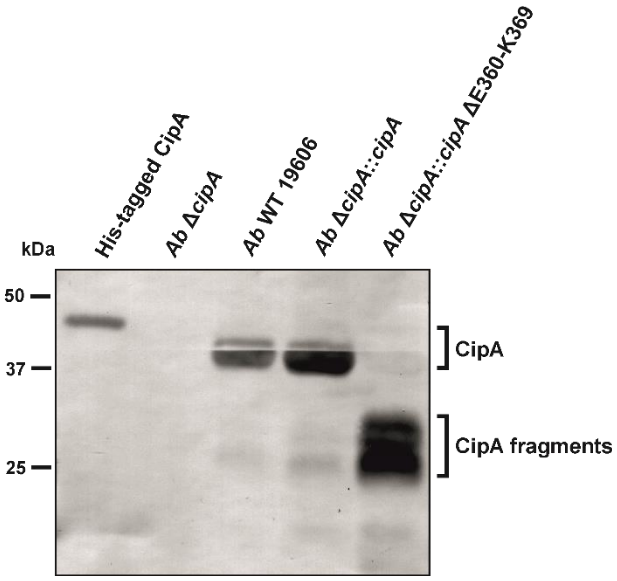

Characterization of *A. baumannii* strains producing diverse CipA variants. CipA was detected in cell lysates (10 µg each) of *A. baumannii* Δ*cipA*, WT 19606 WT, Δ*cipA*::*cipA* and Δ*cipA*::*cipA* ΔE360-K369 by Western blotting using an anti-CipA antibody (1:100). His-tagged CipA was used as control. Native CipA was indicated on the left as well as CipA fragments in strain *A. baumannii* Δ*cipA*::*cipA* ΔE360-K369.
