# Supplementary material for: CipA mediates complement resistance of Acinetobacter baumannii by formation of a factor I-dependent quadripartite assemblage
Source: Front Immunol. 2022 Jul 26;13:942482. doi: 10.3389/fimmu.2022.942482 (PMC9361855; doi:10.3389/fimmu.2022.942482)
Supplement: Supplementary file 8 [file DataSheet_8.pdf]

Supplementary figure 8

Structural prediction of CipA and CipA E360P obtained with AlphaFold2. Structure prediction was created by using the AlphaFold 2 advanced interface with the default settings using the optional “Refine structures with Amber-Relax” option.
