# Supplementary material for: CipA mediates complement resistance of Acinetobacter baumannii by formation of a factor I-dependent quadripartite assemblage
Source: Front Immunol. 2022 Jul 26;13:942482. doi: 10.3389/fimmu.2022.942482 (PMC9361855; doi:10.3389/fimmu.2022.942482)
Supplement: Supplementary file 9 [file DataSheet_9.pdf]

Supplementary figure 9

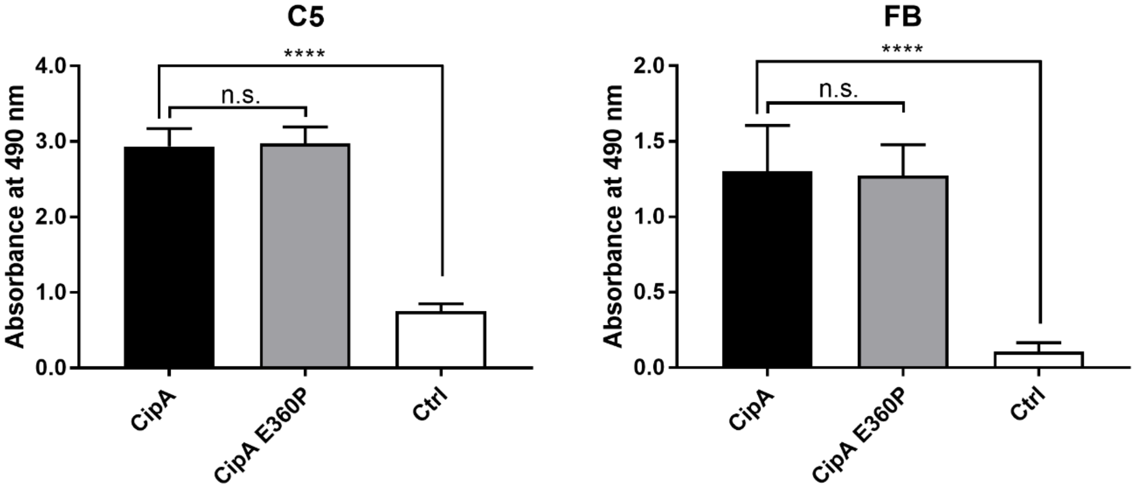

Binding of CipA E360P to C5 and FB. Protein binding of C5 and FB to CipA and CipA E360P was measured by ELISA. Purified bacterial proteins and gelatine (5 ng/µl each) used as negative control were immobilized and incubated with 10 ng/µl C5 or FB. Bound complement components were detected using specific antisera (1:1,000). To assess statistical significance, one-way ANOVA with post-hoc Bonferroni multiple comparison test (confidence interval = 95%) was performed. Data represent means and/or standard deviation of at least three different experiments, each conducted in at least triplicate. ****, $p \leq 0.0001$; n.s., no statistical significance.
