# Supplementary material for: CipA mediates complement resistance of Acinetobacter baumannii by formation of a factor I-dependent quadripartite assemblage
Source: Front Immunol. 2022 Jul 26;13:942482. doi: 10.3389/fimmu.2022.942482 (PMC9361855; doi:10.3389/fimmu.2022.942482)
Supplement: Supplementary file 10 [file Table_1.pdf]

Supplementary table 1. Oligonucleotides used in this study

Oligonucleotide	Sequence (5’-3’)[a]	Use in this work
**Generation of CipA variants with single aa substitutions, deletions in various regions, and extensions**		
CipA-359(-)	TTTTTTAGGCTTTTTGTCGACTTCTTATACAACCATATCTAACACATC	Generation of $CipA_{19-359}$ (aa 19-359), C-terminal deletion of aa 359-369
CipA-145(+)	GCATATTTTCCAAGGATCCAAAGCAACTAATACTCAAGC	Generation of $CipA_{145-369}$ (aa 145-369), N-terminal deletion of aa 19-144
CipA E360A FP	GTGTTAGATATGGTTGTAGCGCAAGAACTCGTTAAAAAAGCC	Generation of variant $CipA_{E360A}$
CipA E360A RP	CTTTTTAACGAGTTCTTGCGCTACAACCATATCTAACACATC	Generation of variant $CipA_{E360A}$
CipA E360P_II FP	GATGTGTTAGATATGGTTGTACCGCAAGAACTCGTTAAAAAGCC	Generation of variant $CipA_{E360P}$
CipA E360P_II RP	CTTTTTAACGAGTTCTTGCGGTACAACCATATCTAACACATCAGC	Generation of variant $CipA_{E360P}$
CipA Q361A FP	GTGTTAGATATGGTTGTAGAGGCAGAACTCGTTAAAAAGCC	Generation of variant $CipA_{Q361A}$
CipA Q361A RP	CTTTTTAACGAGTTCTGCCTCTACAACCATATCTAACACATC	Generation of variant $CipA_{Q361A}$
CipA E362A FP	GATATGGTTGTAGAGCAAGCAACTCGTTAAAAAGCCTAAAAAA	Generation of variant $CipA_{E362A}$
CipA E362A RP	GGCTTTTTAACGAGTGCTTGCTCTACAACCATATCTAACAC	Generation of variant $CipA_{E362A}$
CipA P367A FP	CAAGAACTCGTTAAAAAGGCTAAAAAATAAGTCGACCTGC	Generation of variant $CipA_{P367A}$
CipA P367A RP	GCTTGGCTGCAGGTCGACTTATTTTTTAGCCTTTTTAACGAG	Generation of variant $CipA_{P367A}$
CipA delta L363 FP	GGTTGTAGAGCAAGAAGTTAAAAAGCCTAAAAAATAAGTC	Generation of variant $CipA_{\Delta L363}$ lacking a leucine at aa position 363
CipA delta L363A RP	AGGCTTTTTAACTTCTTGCTCTACAACCATATCTAACAC	Generation of variant $CipA_{\Delta L363}$ lacking a leucine at aa position 363
CipA delta V364 FP	GTAGAGCAAGAACTCAAAAAGCCTAAAAAATAAGTCGACCTG	Generation of variant $CipA_{\Delta V364}$ lacking a valine at aa position 364
CipA delta V364 RP	GAGTTATTTTTTAGGCTTTTTGAGTTCTTGCTCTACAACC	Generation of variant $CipA_{\Delta V364}$ lacking a valine at aa position 364
CipA delta P367 FP	CAAGAACTCTTAAAAAGAAAAAATAAGTCGACCTGC	Generation of variant $CipA_{\Delta P367}$ lacking a proline at aa position 367
CipA delta P367 RP	GCTTGGCTGCAGGTCGACTTATTTTTTCTTTTTAACGAG	Generation of variant $CipA_{\Delta V367}$ lacking a proline at aa position 367
CipA delta L363-V364 FP	GTTGTAGAGCAAGAAAAAAAGCCTAAAAAATAAGTCGACCTG	Generation of variant $CipA_{\Delta L363-V364}$ lacking leucine and valine at positon 363 and 364
CipA delta L363-V364 RP	GAGTTATTTTTTAGGCTTTTTTTCTTGCTCTACAACC	Generation of variant $CipA_{\Delta L363-V364}$ lacking leucine and valine at positon 363 and 364
CipA-364 FP	GTAGAGCAAGAACTCGTTTAATAACCTAAAAAATAAGTCGAC	Generation of variant $CipA_{\Delta V364-K369}$ lacking aa at positions 364 to 369
CipA-364 RP	GTAGAGCAAGAACTCGTTTAATAACCTAAAAAATAAGTCGAC	Generation of variant $CipA_{\Delta V364-K369}$ lacking aa at positions 364 to 369

CipA-L363 FP	GTTGTAGAGCAAGAACTCTAATAAAA GCCTAAAAAATAAGTCGAC	Generation of variant CipA$_{\Delta L363-K369}$ lacking aa at positions 363 to 369
CipA-L363 RP	CGACTTATTTTTTAGGCTTTTATTAGA GTTCTTGCTCTAC	Generation of variant CipA$_{\Delta L363-K369}$ lacking aa at positions 36 to 369
CipA-E362 FP	GTTGTAGAGCAAGAATAATAAAAAA AGCCTAAAAAATAAGTC	Generation of variant CipA$_{\Delta E362-K369}$ lacking aa at positions 362 to 369
CipA-E362 RP	CTTATTTTTTAGGCTTTTTTTATTATTC TTGCTCTAC	Generation of variant CipA$_{\Delta E362-K369}$ lacking aa at positions 362 to 369
CipA-Q361 FP	GGTTGTAGAGCAATAATAAGTTAAAA AGCCTAAAAAAT	Generation of variant CipA$_{\Delta Q361-K369}$ lacking aa at positions 361 to 369
CipA-Q361 RP	GGCTTTTTAACTTATTATTCCTCTACA ACCATATC	Generation of variant CipA$_{\Delta Q361-K369}$ lacking aa at positions 361 to 369
CipA-E360 FP	GATATGGTTGTAGAGTAATAACTCGT TAAAAAGCCTAAAAAAT	Generation of variant CipA$_{\Delta E360-K369}$ lacking aa at positions 360 to 369
CipA-E360 RP	GGCTTTTTAACGAGTTATTACTCTACA ACCATATCTAAC	Generation of variant CipA$_{\Delta E360-K369}$ lacking aa at positions 360 to 369
**Generation of *A. baumannii* 19606 strain producing a CipA variant by markerless mutagenesis**		
CipA up fwd PstI	GCGACTGCAGCAAACTCAGGTTATTG AACTCCCAATGG	Amplification of the upstream region of the CipA encoding gene in strain *A. baumannii* 19606
CipA down rev NotI	GACAGCGGCCGCGGGATTTAATATTT TGCTGCTAAATG	Amplification of the downstream CipA encoding gene in strain *A. baumannii* 19606
CipA V359 RP	GTTAGATATGGTTGTATAGTAATAAC TCGTTAAAAAGCCTAAAAAA	Introduction of two stop codons after position 359
CipA V359 FP	GGCTTTTTAACGAGTTATTACTATACA ACCATATCTAACACATC	Introduction of two stop codons after position 359
CipA control fwd	GCTCTTGTCTATCTTATGTCACAGATA GCC	Control of the complementation in *A. baumannii* 19606
CipA control rev	CTGGCTGTCCACCAGGAACACATTTA TTTGC	Control of the complementation in *A. baumannii* 19606
CipA seq Fwd	CAATCCAAACCAACGCGTAATCTTAC GTG	Sequencing of *cipA* gene in the complemented *A. baumannii* 19606
CipA seq Rev	GGTACTGTGAATTATGTAGATGGTGC C	Sequencing of *cipA* gene in the complemented *A. baumannii* 19606
**Primer used for RT-PCR of *A. baumannii* genes**		
rpoB-RT-fwd	GAGTCTAATGGCGGTGGTTC	RT-PCR, amplification of *rpoB* in *A. baumannii*
rpoB-RT-rev	ATTGCTTCATCTGCTGGTTG	RT-PCR, amplification of *rpoB* in *A. baumannii*
CipA-RT-PCR Fwd	GTCATGTCAACTATGATGGC	RT-PCR, amplification of *cipA* in *A. baumannii*
CipA-RT-PCR Rev	GCAAGCAAGTTTGTGGTGCAAC	RT-PCR, amplification of *cipA* in *A. baumannii*
**Primer used for the generation of His-tagged Efb and Efb-C proteins of *Staphylococcus aureus* USA300**		
Efb_FP Bam	GCGAGCGAAGGATCCGGTCCAAGAG	Amplification of Efb encoding gene of

	AAAAGAAACCAGTGAG	*S. aureus* USA300
Efb_RP_Sal	GGCTGCAG<u>GTCGAC</u>TTATTTAACTAA TCCTTGTTTTAATAC	Amplification of Efb encoding gene of *S. aureus* USA300
Efb-C_FP_Bam	GGTGCA<u>GGATCC</u>CAATTTAATAAACC AGCAGCGAAAACTG	Generation of a C-terminal fragment of Efb
**Primer used for sequencing**		
pQE-FP-30	TTGCTTTGTGAGCGGATAAC	Sequencing of pQE-30 Xa vector
pQE-RP	CTGAGGTCATTACTGGATCTATC	Sequencing of pQE-30 Xa vector

[a], Sequences of specific restriction endonuclease recognition sites are underlined
